# The Action of the Voluntary Muscles

**Published:** 1862-05

**Authors:** 


THE ACTION OF THE VOLUNTARY MUSCLES. An extract of sixteen pages from an unpublished work. By LOUIS MACKALL, M. D., Georgetown, D.C.

In the pages sent us with the above title, Dr. MACKALL advances the doctrine that the *active* state of a muscle is that of *elongation* instead of contraction; thus reversing the mode of action hitherto regarded as fully established.

Thus he says: "The extension of the leg on the thigh is effected by the action of the muscles on the *posterior* surface of the thigh bone or femur, while the leg is flexed on the thigh by the action of the muscles on the *anterior* surface of this bone;" &c. He represents the innervation or active determination of nerve force to a muscle, as producing *erection and elongation* of its fibres; while the withdrawal of nerve force produces contraction and repose in the muscle. To establish these novel doctrines in relation to muscular action will require a much stronger array of facts and experimental demonstrating than are to be found in the pages sent us by Dr. MACKALL.

---

THE LONDON LANCET, for May is, as usual, promptly on our table. It also contains its usual amount of practical and interesting reading matter.

